# Supplementary material for: Are carbon emissions trading and green financial instruments synergistic? -Comprehensive quantitative research based on content analysis
Source: PLoS One. 2024 Mar 7;19(3):e0298601. doi: 10.1371/journal.pone.0298601 (PMC10919601; doi:10.1371/journal.pone.0298601)
Supplement: S1 Data — (ZIP) [file pone.0298601.s001.zip › Data/地级市Z_F工作报告.zip!/╝¬┴╓/╦─╞╜╩╨/└┤╫╘╣1⁄2╓┌║┼í╛┬φ┐╦╩2╛▌═°í┐_2013.docx]

各位代表：

　　现在，我代表四平市人民政府，向大会报告政府工作，请予审议，并请市政协各位委员提出意见。

　　一、2012年工作和过去五年回顾

　　2012年，在省委、省政府和市委正确领导下，在市人大、市政协监督支持下，市政府团结带领全市人民，贯彻“一核三带”富民优先战略，围绕“六增力一改观”目标，实施“四突破五确保”任务，九项重点工作强力推进、硕果累累，四平发生了化蛹成蝶的进步与变化！

　　――抓固定资产投资，项目建设实现突破。通过资源要素的优化配置，促进项目谋划、评估、选商、开工等环节质量实现跃升。强化规划机构职能，发挥规划统筹作用，促进产业和生产力合理布局。在项目增多、用地紧张情况下，集约利用土地资源，规范清理存量，合理使用增量，全市收储土地942.6公顷，其中市本级收储土地643.9公顷，确保重大项目成功落地。完善政府金融职能，发挥银行等金融机构作用，设立我市第一支创业投资基金，引导企业上市直接融资，投融资渠道不断拓展，有效筹集发展资金，获得全国首批金融生态示范城市称号。创新成立五个市级要素配置服务中心，制定10个方面人才培养实施方案，与吉林大学等院校签订战略合作框架协议，成功举行“院士专家四平行”活动，为培育引进人才及智力资源搭建平台。全市亿元以上建设项目270个，其中10亿元以上在建项目达24个。“4·18”新开工亿元以上项目，由4年前的19个，上升至99个。建成全省首家重大项目储备库，滚动储备超亿元项目141个，现已出库开工建设项目19个。组建重点产业课题组，主动与部分央企、民企集团及国内外著名中介机构开展项目对接、投资洽谈和专题招商。项目履约率、投入产出率、净利润率越来越高，项目建设规模、质量、效率实现新突破。

　　――抓产业升级扩能，工业经济增强活力。克服经济下行压力，全力解决工业运行中遇到的新情况新问题，实现工业经济平稳较快发展。开发区、工业集中区发展步伐加快，公主岭经济开发区步入选商、规模发展阶段，双辽玻璃建材园急剧扩张，奇瑞、“一汽四环”、模具等工业园区发展势头强劲，换热器、专用车、汽车零部件等工业园区成为省级特色工业园区。我市成为全省先进装备制造业示范城市，部分产业纳入国家振兴规划，梨树县被列为国家级农副产品新型加工示范区。企业嫁接改造成果显著，成功引进奇瑞重工、中航工业、中国建材、三一重工、柳工、法国飞马特等国内外知名企业。共实施技术改造196项，完成投资239亿元。7项省级重大科技攻关、成果转化等项目落户我市。投产新产品158种，完成产值180亿元。累计获得6项中国驰名商标。单位GDP综合能耗降低3.5%，主要污染物总量减排完成全年目标。

　　――抓结构调整优化，“三经”工作卓有成效。结合区位优势，快速聚集新的经济能量，探索提升服务业发展新途径，以区域总部经济为主导的“三经”工作成为经济发展中的蓝海。大规模集中培训720多人，在13个省、4个直辖市和部分发达地区召开56场推介会，到会客商1.5万人。制定优惠政策，全程跟踪服务，成立四平总部大厦及县（市）区总部服务中心，吸引发达地区企业挥师北上，进军东北，入驻四平。预计到年末，全市发展总部企业520户，新增税收6亿元以上，为财税扩张增添新动力，开辟了四平总量发展的新天地。民营企业达到1万户，同比增加1324户；个体工商户达到13.2万户，同比增加2万户。东北亚农副产品数贸港落户梨树，维美戴斯酒店投入运营，红星美凯龙商城、开元大酒店及城市综合体等服务业项目加速建设。大连万达、中国供销集团、上海亿丰、广东东鹏及天津港、大连港、营口港合作建设的内陆港等一批新兴服务业有望入驻四平。

　　――抓“三区一建”提标，农村形势持续向好。始终把“三农”工作放在重要位置。粮食生产战胜多种灾害，实现“九连丰”，有望实现160亿斤新纪录。“三区一建”提标取得新进展。新建粮食高产示范区101个，率先成为国家级整建制推进市。新建棚室蔬菜园区20个，生产面积达到11.3万亩，应季蔬菜自给率达到90％，返季蔬菜自给率达到20％以上。新建牧业小区324个，总量达到2212个，规模化养殖水平居全省前列。农村人居环境建设力度加大，乡（镇）村屯环境面貌发生根本性改观，各地普遍打造一批环境优、功能全、文明向上的乡（镇）村屯。农产品品牌建设迈出新步伐。一大批具有“三品一标”的名特优农产品打入市场，生产基地进一步扩大。农村改革不断深化，土地适度规模经营、土地承包经营权收益抵押贷款试点、农业社会化服务组织、农村新型社区和农民专业合作社等改革模式正在推进农村改革向纵深发展。

　　――抓基础设施投入，城乡面貌焕然一新。迎接高铁时代到来，东南新城建设步伐加快。高铁客运站在哈大高铁线上靓丽运行，四平东综合客运枢纽站主体竣工，旭日立交桥主桥贯通，城市规划馆、政务中心、公检法办公楼等工程重点推进，启动文化艺术中心、学校、医院、交通指挥中心规划设计工作。老城区梳理改造有序进行。中心城区内环路改造工程开始实施，新华大街段、紫气大路段建成通车，6公里滨河景观路按期交付使用。维修道路16条，新建改造道路桥梁5座，主次街路改造基本完成。高标准实施紫气大路、开运街等街路亮化工程。排水、供热等地下管网及人防空间改造建设大幅拓展。加快保障性住房建设，完成棚改项目46个、357.6万平方米，建设回迁安置用房101万平方米。建设廉租房12万平方米。完成“暖房子”工程80万平方米。春秋两季市区大树进城18万棵，西南生态公园、“两河四岸”、厂区及庭院绿化工程进展顺利，城在林中形态初步显现。更新城市公交车63台。加大城区环境整治力度，国家卫生城复检正式通过。“数字化城市”建设取得积极进展。新建改建农村公路463.6公里。伊通南部新城、公主岭岭西新城、梨树南部新城、双辽辽东新城建设齐头并进，气象日新！

　　――抓百姓期盼之事，民生工作成效显著。年初确定的10个方面35件民生实事全面告捷。全市城镇新增就业6.4万人，农村劳动力转移就业72万人，城镇登记失业率控制在3.8%以内。养老、失业等五大保险参保人数均提前完成年度指标。新农保和城居保参保率达到96%以上，成为全省之最。最低工资标准由每月890元提高到1050元。公务员连续四年提高津补贴标准，增幅列全省首位。四平收入水平节节攀升！城乡低保在动态管理下应保尽保，实现市区居家养老服务全覆盖。“四师一满意”主题教育活动取得阶段成果，67.34万平方米校安工程建设任务基本完成。公共文化场馆全部免费开放，开展送戏下乡365场，成功举办元宵节灯会、“叶赫风情”大型文艺演出，万名中小学生满族花棍舞表演创吉尼斯纪录。开展百姓健康大行动，完成220万城乡居民免费体检，创全国地级市全民免费体检先河。全国公路自行车冠军赛、市直机关第四届职工运动会取得圆满成功，扩大了声誉、提振了士气。全市6个县级食品安全快速检测站和158个快速检测室完成布局并组织实施。扎实推进“社区行动计划”，76个社区全部建立公共事务服务中心，形成就业、社会保障、医疗、计生、文化、教育、安全等进社区服务模式。实现全国双拥模范城六连冠。扎实推进社会管理创新“331”工程，受到全省关注。认真落实责任追究和领导包案制度，集中处理信访积案，确保了重要时点安全稳定。完善治安防控体系，新增50台夜巡警务车，主要街路全部安装电子监控设备，社会面稳控能力提高，治安环境明显改善，可防性案件下降38%。安全生产和应急工作常抓不懈。形成了和谐安定的可喜局面。

　　――抓协调运行机制，政府建设全面加强。坚持在“四型”政府建设上下功夫，狠抓各项工作落实，提升政府执行力。坚持定期向市委报告制度，自觉接受人大法律监督和政协民主监督。坚持重大事项集体决策制度。实行政府信息公开，通过政务大厅、政府网站、新闻媒体、新闻发布会等形式，加强与群众沟通联系，提高办事服务效率。对政府工作实况实行日报告、周安排、月通报制度，督促各部门集中力量抓重点工作，并定期公示考核结果，好的表彰奖励，差的通报批评。深化行政审批制度改革，所有行政审批项目实现“一站式”办理，审批效率大幅提升。事业单位清理规范工作任务圆满完成。深入开展效能监察和审计监督，加强重大项目全程跟踪，严肃查处违法违纪案件。要求机关干部读好市场营销、企业管理、金融证券、科技创新四本书，以增强经济工作才智。全面落实党风廉政责任制，自觉遵守领导干部廉洁自律规定。普法、民族宗教、档案、地震、老龄、残联、地方志、外事侨务等工作取得进步与发展，驻平中省直部门发挥效能，为四平建设发展做出了新贡献。

　　在看到成绩的同时，我们也清醒地认识到，前进中还存在一些困难和问题。产业层次不高，结构调整任重道远；受经济下行压力等因素制约，致使部分经济指标受到较大影响，财政收入未能达到奋斗目标；政府融资受限，基础设施建设资金短缺；工业经济实力还不强，缺乏税源型龙头企业；公务人员市场意识、创新意识还需强化。我们要高度重视，采取措施，认真加以解决。

　　各位代表，即将过去的五年，是四平发展史上不平凡的五年，是综合实力提升最快、城乡面貌变化最大、改革开放成效最好、人民群众受益最多、对内凝聚力和对外影响力显著提升的重要时期。

　　――2012年预计实现地区生产总值1186亿元，是2007年的2.5倍，五年年均增长20.1%。地区生产总值突破千亿元大关，成为我市发展进程中的里程碑！

　　――2012年预计全社会固定资产投资600亿元，是2007年的2.7倍，五年年均增长22.1%，增幅连续五年居全省第二位。

　　――2012年预计规模以上工业总产值、增加值、利润分别完成1700亿元、520亿元和51亿元，是2007年的4.5倍、4.3倍和4.6倍，五年年均增长35.2%、33.7%、35.9%，均跃升至全省第四位。

　　――2012年预计全口径财政收入、地方级财政收入分别完成95.7亿元、50.5亿元，是2007年的3.3倍、4.1倍，五年年均增长27.1%、32.5%。财政收入占GDP比重逐年提高。2012年预计市本级地方级财政收入22.8亿元，是2007年的4.5倍，五年年均增长35%， 2010、2011两年实现翻番，总量由全省第八位跃升到第四位，开始走出财力弱、负担重、艰难跋涉的困境。

　　――2012年预计全市财政支出163亿元，是2007年的3.2倍，五年年均增长26%；市区财政支出58亿元，是2007年的3.5倍，五年年均增长28.1%。

　　――2012年预计市区向上争取专项资金28亿元，是2007年的4.3倍，五年年均增长34%。

　　――2012年预计实现社会消费品零售总额395亿元，是2007年的2.5倍，五年年均增长19.9%。

　　――2012年预计金融机构存贷款余额分别达到745亿元、438亿元，是2007年的2.3倍和1.5倍。

　　――2012年预计城镇居民人均可支配收入、农村居民人均纯收入分别实现2万元、8958元，比2007年增长62.2%和109.3%，五年年均增长10.2％、15.9%。

　　――2012年，在全国294个城市中，四平列竞争力提升最快城市第32位，四平声音与地位显著提升！

　　成绩来之不易，经验弥足珍贵。五年来，我们深深地体会到：

　　观念转变是发展之先导。围绕加快转变、加快赶超、加快隆起，打破计划经济的思维定势、固步自封的观念束缚和小富即安的小农意识，谋划发展更加注重效益意识、资本意识、科技意识和市场营销，尊重纳税人和企业家，尊重创新发展人才。更加注重从规划上确立目标，在资金、人才、市场等要素和攀高结贵上确定支撑，更加注重优胜劣汰、奖优罚劣，形成抓项目促发展的“群狼效应”。转变观念、清晰路径、形成机制，推动经济合力前行，四平迎来了发展建设的春天！

　　科学规划是发展之基础。立足落实“一核三带”富民优先战略，构筑经济发展与生产力空间布局。第一次完整编制城市总体规划、土地利用总体规划、东南生态新城规划、“两河四岸”景观建设规划、四大特色产业园区规划和城市专项规划、重大项目规划。中心城区控详规覆盖率达到100%。城市路网骨架不断拓展，主要道路零标高以下良心工程高质量完成，城市南部横向景观轴业已形成，成功创建国家园林城市。一座特色鲜明、具有都市形态的新四平正在屹立之中！

　　目标路径是发展之要法。摒弃单一本位工作思路，鼓励支持外出学习先进经验理念，引进国内外专业机构，咨询评估发展规划战略。以投入产出率评估项目、配置资源、倾斜政策、研究发展关节点。把破解资金瓶颈落实到财政增收、向上争取专项、上市融资、发展总部经济等措施上，筹措发展资金，加大投资强度。在企业嫁接改造、大树进城、发展总部经济、滨河景观绿化带、全民免费体检等方面，实现目标延伸、路径确认、方案制订、破解难题与高标准推进紧密结合，探索克难攻坚的新路径，值得珍惜弘扬。四平发展的正能量，正在全面振兴的征程上积聚！

　　实践创新是发展之灵魂。把开放发展放在首位，迈开大步走出去，足迹遍布大江南北，与国内外的交流合作日益加深。分别与长春、沈阳及部分央企签署战略合作框架，四进欧洲寻求合作机遇，专用车欧盟路演受到青睐。烟厂实施易地改造，奇瑞重工成功落地，国内外30多家知名企业入驻四平。共谋发展请进来，成功举办换热器国际研讨会、四平·中国汽车拉力锦标赛、首届中国玉米节等盛会，四平音符和影响力日益增强。全国生态环境建设实验区、国家可持续发展实验区、国家循环经济示范区创建启动，红嘴开发区晋升为国家级开发区，搭建起率先发展平台。下决心规范清理机构编制，首次实现编制人员“负增长”。加快推进阳光政务，注重执行落实和提高效率，经济发展软环境由全省末位跃升至前列。广大干部谋发展、促振兴的热情持续高涨，众人划桨开大船的动力机制业已形成！

　　各位代表，过去五年，勤劳智慧的四平人民，积极抢抓发展机遇，有效应对各种挑战，砥砺奋进，铿锵前行，用激情、拼搏和奉献，谱写了四平科学发展、攀升晋位的辉煌篇章！成绩的取得，得益于市委的坚强领导和历届班子打下的坚实基础，得益于人大、政协和社会各界的鼎力支持，归功于全市人民的群策群力。在此，我代表四平市人民政府，向全市各族人民、各民主党派、工商联和无党派人士，向驻平部队、武警官兵、中省直单位，向所有关心支持四平改革发展的同志们、朋友们，致以崇高的敬意和衷心的感谢！

　　二、今后五年的目标任务

　　未来五年，坚持把市六次党代会确定的指导思想、目标任务作为工作统领。保持清醒头脑，顺应形势发展的新变化，顺应全市人民过上更加美好生活的新期待，抢占发展制高点，打造振兴隆起带，开创科学发展、富民强市的新局面。

　　未来五年，政府工作的总体要求是：高举中国特色社会主义伟大旗帜，深入贯彻落实党的十八大、省十次党代会和市六次党代会精神，以“科学发展，努力让城乡居民生活得更加美好”为统领，纵深推进“一核三带”富民优先战略，坚持“四动”并举，推进“四化”统筹，紧紧围绕“五城联创”，加快经济发展方式转变，加强社会管理创新，推进项目大建设、产业大集聚、城乡大改观、文化大繁荣、民生大改善，全面提升城市核心竞争力、文化软实力和区域影响力，加快在哈大经济大动脉上的强势隆起，实现四平全面振兴。

　　按照市委要求，未来五年的奋斗目标是：一跨越、两翻番、双倍增、创五城、五提升。

　　一跨越——五年内，综合经济实力跨入全省第一方阵。

　　两翻番——到2017年，地区生产总值在2012年基础上翻一番，达到2370亿元以上；全口径财政收入和地方级财政收入在2012年基础上翻一番，分别达到210亿元和105亿元以上。

　　双倍增——到2017年，城镇居民人均可支配收入、农民人均纯收入在2012年基础上增加一倍，分别达到4万元和1.8万元以上。

　　创五城——创建区域中心城、新型工业城、特色文化城、现代宜居城、富裕文明城。

　　五提升——发展质量提升、城市化水平提升、文化实力提升、幸福指数提升、社会管理创新提升。

　　未来五年，我们必须把市六次党代会确定的“五城联创”作为富民强市“路线图”，推进市区率先发展、县市联动跟进，求得全局实现新跨越。

　　(一)打造活力迸发的区域中心城。深入实施“一核三带”富民优先战略，筑起现代化大城市骨架，打造百万人口的区域中心城市。加快“南接北融”步伐，主动承接长春、沈阳发展的乘数效应。盯住国内外500强、央企和大型民企主动招商、承接项目，着力提高发展外向度。继续发展总部经济，建设中小企业创业园，深入开展“院士专家四平行”活动，让更多智力资源、科技成果汇聚四平。发挥区位优势，发展生产性服务业，做强做活商贸物流业，打造辐射东北四省区的商贸物流汇聚地。引进域外金融机构来平共建融资平台，规范发展各类金融机构，鼓励更多企业上市，形成资金洼地效应。借助高铁开通契机，加快高速公路、一级公路、铁路建设步伐，推进军民合用机场通航，构筑与周边经济体一小时通达的立体交通体系。把环境作为要素流入的第一竞争力，改革管理体制，完善长效机制，建设区域一流软环境，让人流、物流、资金流、信息流等要素汇聚四平，全面提高开放型经济水平，把四平打造成充满活力的新型现代化城市！

　　（二）打造特色鲜明的新型工业城。积极争取成为“全国老工业基地调整改造规划”重点城市。围绕装备制造、农产品加工和能源三个主导产业，打造中国换热器城、北方专用车基地、一汽零部件配套中心、东北“糖都”和绿色农副产品精深加工区。能源产业实现突破，医药化工、矿产建材等传统产业改造提升，精心打造“烟酒食糖”、“机油器车”特色优势产业，支持鼓励金士百、新天龙、巨元、吉林世宝、一汽专用车等企业兼并重组、引进战投、合资合作，扩大企业规模，尽快形成一批企业集群。实施创新驱动战略、名牌带动战略，加快新产品、新技术和科研成果转化项目开发与引进，支持鼓励九洲光电、神韵超薄音响等科技创新型企业发展，争创全国质量强市示范城市，提升核心竞争力。加快特色园区建设，现有省规划管理园区全部晋升为省级特色园区。支持开发区和工业集中区率先发展、跨越发展、集聚发展，县（市）区积极培育改革创新发展先行区、转型升级示范区、“三区一建”示范区。提升农业综合生产能力，加快基础设施和新农村建设，推进城乡发展一体化，形成新型工农关系。让工业化、信息化、城镇化和农业现代化“四化”并举的发展格局早日在四平大地呈现！

　　(三)打造生态和谐的现代宜居城。以生态文明建设为目标，推进经济社会发展总体规划、城市总体规划、土地利用总体规划、“十二五”林带总体规划、水利用总体规划、交通总体规划“六规统一”，构建统筹协调发展格局。加快发展核心城区，筑起现代大城市骨架，打造百万人口的区域中心城市。全面建成东南生态新城，启动开发北部新城。完善扮靓提升老城区，推进企业退城进区，引入连通城市水系，综合开发“两河四岸五湖”，巩固扩大国家卫生城、园林城成果。积极建设“智慧城市”，推进城市公共事业提升。加快城市基础设施建设，推进城市道路通达工程建设。优先发展城市公共交通。推进四县（市）新城建设，公主岭建成中等城市；梨树加快与四平同城化发展；伊通迈向全国一流少数民族自治县行列；双辽打造省能源基地、区域性中心城市；铁东建成新型工业化基地与旅游文化强区；铁西全力打造现代服务业强区；辽河垦区打造商贸流通“旺地”。同时，围绕“一核三带”节点布局，培育一批工业强镇、商贸大镇、旅游名镇。促进生产空间集约高效、生活空间宜居适度、生态空间山清水秀，建设生态四平、绿色四平、美丽四平！

　　(四)打造底蕴深厚的特色文化城。围绕历史传承、资源特色，形成“一主四辅”的文化发展新格局。大力弘扬社会主义核心价值体系，以四平精神为引领，广泛开展文明创建活动，力争早日进入全国文明城市行列。加快文化基础设施建设，实现市有文化中心、县有文化馆、乡有文化站、村有文化室。推进“文化七进和三下乡”、“九月鲜花”、农村电影放映等文化惠民工程，继续举办叶赫风情大型文艺演出、元宵灯会、“广场之夏”等活动，积极争取举办全国少数民族文化周。挖掘红色文化、创业文化、工业文化、农耕文化、满族民俗文化资源，积极申报和建设省历史文化名城。继续深化文化体制改革，培育文化“核心层”企业，推动文化产业跨越发展。加强文化人才队伍建设，鼓励文艺产品创作，造就一批精品力作和名人名家，增强文化创造活力。创建文化强市，构建起引领风尚、吸引群众、服务社会、推动发展的四平人民的精神家园！

　　(五)打造惠民利民的富裕文明城。围绕学有所教、劳有所得、病有所医、老有所养、住有所居，统筹发展与民生，实现居民生活品质与经济发展同步增长。通过综合施策，推动城乡居民更高质量就业和全员创业。引导群众理性投资，不断增加财产性收入。建立机关事业单位工资正常增长机制，逐步提高企业最低工资标准，实现城乡居民收入5年倍增目标。统筹推进城乡社会保障体系建设，健全全民医保体系，实现人人享有基本社会保障。深入开展“社区行动计划”，统筹城乡社区建设，提高公共服务能力。加快廉租房、公租房、经济适用房建设和农村危旧房改造，全面完成“暖房子”和棚户区改造。加大扶贫开发力度，实现扶贫对象早日脱贫致富。推动教育优先发展、均衡发展、公平发展，调整优化教育结构，大力发展职业教育，提高教育现代化水平。加快医疗卫生事业发展，不断改善提高医疗环境和医护水平，建成覆盖城乡的标准化医疗卫生机构，提升人民健康水平。开展全民健身活动，提高竞技体育水平，完善体育基础设施，积极承办体育赛事。完善提升广播电视节目质量，实现全市广播电视无缝覆盖。深入开展生育关怀和创建幸福家庭活动，继续把四平计生品牌推向全国。加大安全生产保障投入，推进隐患排查治理体系建设。食品药品安全监管体制机制更加完善。加强和创新社会管理，完善矛盾纠纷排查调处、社会稳定风险评估、公共安全管理、应急管理等机制，构建和谐稳定新格局。让四平人民过上更加富裕快乐、和谐尊严、健康舒适的美好生活！

　　未来五年，我们落实市六次党代会精神，真正提升政府执行力，还必须树立市场经济意识，从体制机制上实现根本转变。一是完善竞争机制。通过绩效考核，创建奖勤罚懒、优胜劣汰的工作环境。强化效能驱动，最大限度地调动每个人的积极性，打造重点工作执行、监督、考评、奖惩一体化的“落实链条”。通过引进、选拔、使用和待遇等措施相结合，优待人才、鼓励先进，激发内在动力，形成“百舸争流”、共谋发展的强大合力。二是实现效益配置。下力量把科技、资源、成本等要素优化组合，实现效益最大化。确保行政行为与市场行为相一致，对公共管理和资源配置，严格成本核算，计算投入产出比，用最合理的投入为群众办更多实事。严格按未来收益评判项目，以市场导向、附加值、净利润率和发展前景作为标准，集约使用资源，提高发展的持续性。三是形成开放格局。切实把走出去请进来战略落到具体工作中，向前推进迈大步。破除计划经济的思维定势和封闭意识，把四平融入到全球经济一体化之中，做到外资、外贸、外经、外包、外智“五外”联动。加强与央企、央院、央校、金融机构和知名民企战略合作，引导企业不求所有但求发展壮大，创新开放模式，完善开放型发展体系。四是营造法治环境。坚持依法行政，加强政府引导调控、扶持服务、监管规范职能作用，为市场主体——企业创造良好发展环境。鼓励企业发展壮大，减少运行成本，帮助企业家拓宽视野，提升经营能力。严格按照法律法规、市场规律和国际规则办事，做到讲制度、重程序、守纪律、强监督。

　　各位代表！经过全市上下五年的不懈努力，我们一定能实现赶超跨越，一定能在哈大经济大动脉上强势隆起，四平的未来会更加强市富民、璀璨夺目！

　　三、2013年主要工作安排

　　2013年是新一届政府履职的第一年，也是全面贯彻落实党的十八大精神的开局之年，经济社会发展具备很多有利条件和积极因素。同时必须增强忧患意识和紧迫感，充分估计面临的困难和挑战，开拓创新，扎实开局。主要预期目标是：地区生产总值增长15％以上；固定资产投资增长30％以上；全口径、地方级财政收入增长18%和20%；社会消费品零售总额增长20％；城市居民人均可支配收入、农村居民人均纯收入分别增长11％以上；居民消费价格总水平控制在3%左右；城镇登记失业率控制在4%以内。重点做好以下工作：

　　（一）着力转型升级，实现工业经济平稳较快发展。加快传统产业改造步伐，推动新兴产业、先进制造业健康发展，提高企业核心竞争力。加快落实我市纳入全国老工业基地改造计划。突出抓好嫁接改造。在摸清企业资产、资源以及清产核资基础上，围绕重点产业和骨干企业，研究市场、技术、销售等行业现状，拿出嫁接改造实施方案。遵循资源变资本、资本变资金的思路，整合包装资源，搭建抵押融资平台，多渠道多方式吸引资金流入四平。依托企业招商引资，与国内外行业龙头企业挂靠联合、战略重组。正在联系洽谈的合作项目，明确领导责任，组织强力推进，务必取得实效。集中力量加快园区建设。各级开发区、工业集中区，全面完成控详规、土地整理、环评等前期工作，利用多种方式推进基础设施建设，加快化工园区、汽车园区二期、物流园区建设，吸纳重大项目，减少前期成本，缩短投入产出周期。积极引进高端专业人才，提供优厚待遇，引进实用人才，落实双重身份，直接服务企业，推动工业企业全面转型升级。

　　（二）着力政策服务，深入推进“三经”工作。继续强力推进总部经济发展。制定优惠政策，开发建设总部基地平台，吸引国内外大型企业集团来平建立分公司、办事处等机构。收集筛选各类行业10大名牌产品，通过全国性行业协会，吸引企业来平建立生产基地和东北经销中心。力争引进10个名牌产品。引进大型商贸物流企业，搭建电子商务平台，建立起面向东北的现代物流网。明确落实部门责任，把合作意向变成落户成果。总部经济形成税收15亿元。大力发展民营经济。落实政策扶持和财政支持，完善中小企业服务体系，培育规模民营企业。采取政府补助、融资担保、规费减免等措施，扶持硕博毕业生等人才来平创业，推进智力小微企业发展。加快发展服务经济。大力发展服务外包、文化创意、现代设计等现代服务业，建设“智慧四平”。挖掘人文资源，开发历史文化街区，建设叶赫满族风情园区和叶赫生态博物馆，打造具有四平特色的旅游业。

　　（三）着力示范引领，大力发展现代农业。围绕“一核三带”节点镇、沿线村，集中连片规模打造粮食高产示范区、棚室蔬菜产业园区、牧业养殖小区、休闲农业体验区，壮大新农村建设样板村群，培育农村经济与社会发展典型。突出品牌农业、效益农业建设，发展特色规模农业、绿色生态农业，扶持专业大户。引进培育龙头企业，发展壮大产业集群，提高农业产业化水平。加强农村基础设施建设，稳步推进农村新型社区和“大村庄”建设，着力发展林牧业，扩大水利建设规模，强化农村电网改造，特别注重县、乡、村道路建设，形成贯通顺畅的交通体系，全面改善农村生产和农民生活条件。深化农村改革，积极推进土地规模经营，扶持发展农民专业合作社、家庭农场，培育发展农业社会化服务体系，支持发展村镇银行，创新农村金融产品，破解农民贷款难、融资难等瓶颈问题，探索新农村建设的新路子。

　　（四）着力梳理改造，建设现代宜居城市。重点实施山、水、林、路、网等方面的改造提升。组织实施重点污染企事业单位退城进区工程，编制北山绿化美化规划。北河开工建设生态防洪工程，与湿地建设相结合，再造草地、沼泽、储水湖等生境构成，形成生态缓解区。继续开展大树进城活动，启动育林基金，确保完成植树造林任务。全面启动城市通达工程，建设简易立交桥、返回式环岛以及过街天桥，形成良性循环的路网系统。在建设维修道路同时，新建、改造燃气管网50公里，同步完成给排水、通讯等地下管网建设。继续加快东南新城建设。“八馆一中心”等公共设施项目全部开工，绿化、亮化、道路等基础设施全面完成，回迁安置房、医院、学校等民生工程快速推进。加大新城收储土地力度，实现转让高效利用，提高商业项目比重。加快政府融资步伐，力争15亿元城投债券按期募集，为城市布局与发展聚集所需资金。

　　（五）着力体制创新，推进民生实事落实。探索改革城区与社区管理体制，把涉及为群众服务的管理职能下放到社区，形成1平方公里左右的管理区间和网格化管理格局，增强社区服务功能。继续抓好衣食住行乐、教育、医疗、就业、养老、和谐十项民生实事。推进高、中、普商业网点合理布局，方便群众购物。发挥食品安全快速检测站及快速检测室作用，实现乡镇以上大型农贸市场检测监督全覆盖，确保食品安全。建设棚改房屋40万平方米、廉租住房10万平方米，改善困难群众的居住条件。四平东综合客运枢纽站和四梨大街竣工交付使用。加快推进城市公交发展，更新车辆131台，开通紫气大路公交线路。推进出租车进档升级。举办文化体育赛会，开展全民健身运动和文体活动，丰富群众文化生活。打造诚信计生，建设幸福家庭。深入开展“四师一满意”主题教育活动，推进教育均衡发展，推动职业教育集团化，努力办好人民满意教育。深入开展“创名院、建名科、树名医、评名护”活动，全面提升医疗水平和服务质量。继续推进百姓健康大行动，完成341万城乡居民免费体检。通过强化培训、增加贷款等综合措施，扎实推动全民创业。建立养老服务社会化体系，增加生活和运营补贴，让老年人生活更有保障。加强治安防范和安全生产工作，妥善解决和面对群众诉求，确保社会安全稳定，努力为广大人民群众创造更安宁、更幸福、更欢乐和谐的社会环境！

　　各位代表，在更高起点上实现新跨越，需要强化“四型”政府建设，尤其注重懂经济、讲服务、重结果、守规矩的落实，切实增强公信力和执行力。

　　把为民服务作为政府的第一目标。坚持人民利益至上的价值标准，实实在在地为企业纳税人服务、为群众服务、为基层服务。大力精简审批事项，深入实施投资项目并联审批，努力创造在全省审批项目最少、程序最简、费用最低、服务最优、效率最高的服务环境。积极创新政务服务方式，降低行政成本，推进电子政务、网上办公，加强政务大厅、政府网站、市长公开电话、信访接待等平台建设，促进政务服务更加便民、务实、高效。

　　把聚能提效作为政府的运行准则。把竞争机制、效益机制、开放机制、法治机制贯穿于谋发展、抓落实、求结果的全过程。细化工作方案，明确路径，破解难题，迅速打开局面，取得预期效果。强化绩效考核，定期通报结果，以发展论英雄，以贡献定奖赏，激励先进、施压落后。空谈误事，实干兴业。切实做到减少应酬、减少形式、减少坐而论道，腾出更多时间抓项目建设、抓经济发展、抓凝聚人心，多做打基础、利长远的大事，多做解民忧、惠民生的实事。

　　把法治廉洁作为政府的施政之本。严格按照法定权限和程序行使权力、履行职责，依法接受人大及其常委会的法律监督、工作监督，主动接受政协的民主监督，提高人大代表议案、建议和政协提案办理质量。进一步加强行政执法监督，摒弃遏制不作为、乱作为、慢作为现象。全面落实党风廉政建设责任制，推进惩治和预防腐败体系建设，严格落实中央八项规定，加强行政监察、审计监督，主动接受群众监督、舆论监督，树立廉洁、高效、务实、为民的阳光政府形象。

　　各位代表，新的征程已经开启，新的目标催人奋进。让我们在市委的正确领导下，紧紧依靠全市人民，继往开来、凝聚力量、攻坚克难，为实现四平的复兴与发展，为城乡居民生活得更加美好而团结奋斗！
